# Diagnostic accuracy of the PMcardio smartphone application for artificial intelligence–based interpretation of electrocardiograms in primary care (AMSTELHEART-1)

**DOI:** 10.1016/j.cvdhj.2023.03.002

**Published:** 2023-04-05

**Authors:** Jelle C.L. Himmelreich, Ralf E. Harskamp

**Affiliations:** Department of General Practice, Amsterdam UMC location University of Amsterdam, Amsterdam, Netherlands; Personalized Medicine, Amsterdam Public Health, Amsterdam, Netherlands

**Keywords:** Electrocardiogram, Smartphone, Atrial fibrillation, Cardiac arrhythmia, Primary care, Digital health

## Abstract

**Background:**

The use of 12-lead electrocardiogram (ECG) is common in routine primary care, however it can be difficult for less experienced ECG readers to adequately interpret the ECG.

**Objective:**

To validate a smartphone application (PMcardio) as a stand-alone interpretation tool for 12-lead ECG in primary care.

**Methods:**

We recruited consecutive patients who underwent 12-lead ECG as part of routinely indicated primary care in the Netherlands. All ECGs were assessed by the PMcardio app, which analyzes a photographed image of 12-lead ECG for automated interpretation, installed on an Android platform (Samsung Galaxy M31) and an iOS platform (iPhone SE2020). We validated the PMcardio app for detecting any major ECG abnormality (MEA, primary outcome), defined as atrial fibrillation/flutter (AF), markers of (past) myocardial ischemia, or clinically relevant impulse and/or conduction abnormalities; or AF (key secondary outcome) with a blinded expert panel as reference standard.

**Results:**

We included 290 patients from 11 Dutch general practices with median age 67 (interquartile range 55–74) years; 48% were female. On reference ECG, 71 patients (25%) had MEA and 35 (12%) had AF. Sensitivity and specificity of PMcardio for MEA were 86% (95% CI: 76%–93%) and 92% (95% CI: 87%–95%), respectively. For AF, sensitivity and specificity were 97% (95% CI: 85%–100%) and 99% (95% CI: 97%–100%), respectively. Performance was comparable between Android and iOS platform (kappa = 0.95, 95% CI: 0.91–0.99 and kappa = 1.00, 95% CI: 1.00–1.00 for MEA and AF, respectively).

**Conclusion:**

A smartphone app developed to interpret 12-lead ECGs was found to have good diagnostic accuracy in a primary care setting for major ECG abnormalities, and near-perfect properties for diagnosing AF.

**Key Findings**

• In this first independent validation of PMcardio’s electrocardiogram (ECG) interpretation functionality, the application showed excellent accuracy for diagnosing atrial fibrillation, and good accuracy for any major ECG abnormality, among consecutive elderly primary care patients undergoing routine care 12-lead EGC for any indication.
• Diagnostic accuracy for indications of (past) ischemia was more modest, with false-negatives mostly due to the application not acknowledging pathologic Q waves, potentially owing to the algorithm’s training in a higher-risk dataset.
• Accuracy for ischemic ECG markers was higher among the subset of patients presenting for cardiac symptoms, warranting further validation in higher-risk samples.
• Limitations of this validation analysis were its sample size and the low rate of ECG abnormalities in this sample of consecutive primary care patients, limiting its generalization to relatively low-risk settings.

## Introduction

Patients often consult their general practitioner (GP) with symptoms that may be due to an underlying cardiac condition.^1^, ^2^, ^3^ Symptom manifestations include palpitations, chest pain, light-headedness, (near) fainting or dyspnea, and account for 0.8%–16% of symptoms that prompt patients to visit their GP.^1^, ^2^, ^3^ When such symptoms are present, a 12-lead electrocardiogram (ECG) is indicated as part of the diagnostic work-up, a service that most GP practices provide.^4^, ^5^, ^6^ Sometimes, ECGs are easy to interpret; however, when possible abnormalities are observed it may become difficult for less experienced ECG readers to adequately interpret the ECG.

On this background, the PMcardio smartphone application (Powerful Medical, Bratislava, Slovakia) was developed. With the app, operable on Android and iOS platforms, physicians can make a photograph of a 12-lead ECG, which is subsequently analyzed by the in-built artificial intelligence (AI) algorithm. Trained on an existing database of previous ECGs, the app provides an interpretation of the ECG in question, diagnosing abnormalities ranging from arrhythmias to conduction delays to signs of (acute) cardiac ischemia. When also given patient information such as sex, age, reason for presentation, and medical history elements, it subsequently provides tailored advice for (diagnostic) work-up based on local guidelines and protocols. The app is certified for use in Europe (CE, Class II(b) EU MDR medical device) and has a user base of >10,000 physicians across Europe.^7^ However, to our knowledge, PMcardio’s ECG interpretation functionality has not yet been independently validated in a primary care setting. We therefore set out a multicenter validation study in primary care to assess the validity of the PMcardio app as a point-of-care tool for interpretation of routine primary care 12-lead ECGs, using blinded cardiologist interpretation as a reference standard. We hypothesized that the PMcardio app can accurately detect clinically significant ECG abnormalities, irrespective of the smartphone’s platform (and camera) or 12-lead ECG configuration.

## Methods

We reported this diagnostic accuracy study in accordance with the Standards for Reporting Diagnostic Accuracy Studies (STARD) statement.^8^ The study protocol was approved by our institution’s Medical Ethical Review Committee, with further data gathered under the Medical Research Involving Human Subjects Act (WMO) allowing for the use of de-identified retrospective routine care data for research purposes.

### Study design

We used data of consecutive patients that were enrolled in the Validation of a Mobile Bedside ECG Screening and Diagnostic Tool for Arrhythmias in General Practice (VESTA) study (n = 223; April 2017–July 2018), as well as an extension study (n = 72; January–December 2022).^9^ Eligible patients were aged 18 or older who were assigned to 12-lead ECG as ordered by their own GP for any routine care indication in 1 of 11 participating general practices across the Netherlands. The authors performed an independent investigation; the manufacturer of the investigated medical application was not involved in the design, conduct, or reporting of this work.

### Data collection

The study investigators visited participating practices to collect the 12-lead ECG recordings (as PDF file or photocopy of paper original), as well as patient data at time of the index ECG from the practice’s electronic health records. Baseline data included sex, age, indication for undergoing 12-lead ECG, use of relevant cardiovascular medications, and relevant medical history.

### Index test

**Figure 1:**
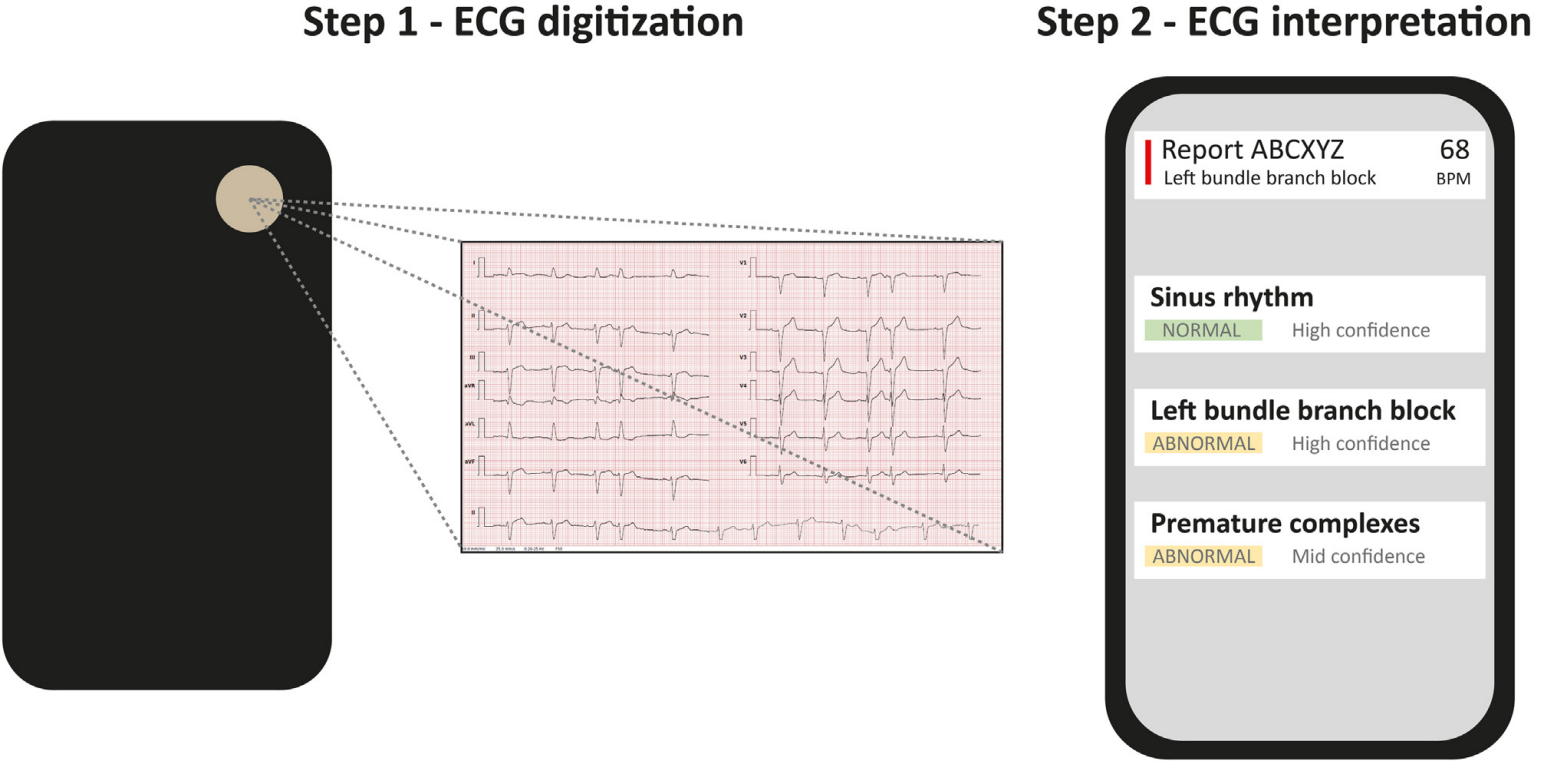
Schematic display of the PMcardio (Powerful Medical, Bratislava, Slovakia) analysis process employed in the current analysis. The figure shows the steps in the PMcardio process employed in the current analysis, ie, electrocardiogram (ECG) digitization by photographing the digital image of a 12-lead ECG on a computer monitor using the PMcardio application (step 1), followed by automated ECG interpretation by PMcardio, the outcomes of which are immediately displayed in the application, along with a confidence level for each detected ECG item (step 2). Note that the PMcardio application also allows the operator to insert reported symptoms and clinical patient data followed by treatment recommendations, as well as the ability to share the report with other medical professionals. The latter steps were outside the scope of the current analysis. ECG interpretation (step 2) is independent from inserted clinical information, which are used only to guide treatment recommendations.

The PMcardio is a smartphone application that allows the user to select a preselection of 12-lead ECG configurations (for instance, 3 rows of 4 leads with 1 rhythm strip [RS] below) and to subsequently enter patient-specific information, as well as a photograph of a 12-lead ECG. The digital image of the ECG is de-identified and encrypted and sent to a central server, where algorithms process the digital image of the ECG. The underlying AI is trained on proprietary clinical databases, and the algorithms can detect 42 distinct ECG features (38 diagnoses and 4 axes, listed in Supplementary Table 1). The response is returned back to the smartphone app (usually within seconds), and the response is reported per abnormality and by a level of confidence (high, mid, or low). We used PMcardio Version 2.5 for the current analyses. A schematic display of the steps employed in the current analysis is provided in Figure 1.

### Reference standard

All 12-lead ECGs were independently evaluated by 2 expert readers (cardiologists or GPs) and, in case of disagreement, by a third reader (cardiologist). We presented the 12-lead recordings in randomized order, and readers were blinded to the results of the index test.

### Outcome definitions

The primary outcome was any major ECG abnormality (MEA) clinically relevant for primary care, which we defined as the composite of atrial fibrillation or flutter (AF), pathologic Q waves, ST elevation or depression, T-wave inversion, high-degree atrioventricular block, left bundle branch block (LBBB), bifascicular block (BFB), trifascicular block (TFB), prolonged QT interval (defined as corrected QT interval >480 ms), or narrow (≤ 120 ms) or broad (>120 ms) complex tachycardia.

Key secondary outcomes were (1) AF; (2) clinically relevant impulse or conduction abnormalities, defined as suspected accelerated junctional rhythm, LBBB, right bundle branch block, BFB, TFB, high-degree atrioventricular block, or prolonged QT interval; and (3) signs of past or present myocardial ischemia, defined as Q waves, ST elevation or depression, or T-wave inversion.

Secondary outcomes were (1) significant ST deviation, defined as ST elevation or ST depression; (2) left ventricular hypertrophy (LVH); (3) ectopy; and (4) any bundle branch block (BBB), defined as LBBB, right BBB, BFB, or TFB.

Presence of each of the outcomes categories was as per consensus by the expert readers, based on current clinical definitions.^10^^,^^11^ Supplemental Table 2 shows the reference ECG abnormalities and corresponding PMcardio categories in each outcome definition.

### Statistical analysis

Diagnostic accuracy was expressed as sensitivity, specificity, positive and negative likelihood ratio (LR+ and LR-, respectively), and positive and negative predictive value (PPV and NPV, respectively) with their 95% confidence interval (95% CI). For sensitivity, specificity, PPV, and NPV, a point estimate of 100% indicates perfect diagnostic accuracy while 0% indicates no predictive ability for the outcomes of interest. For likelihood ratios, LR+ >10 or LR- <0.10 are generally regarded as indicating good test properties for ruling in or ruling out an outcome of interest, respectively.^12^

The primary analysis of this study was to assess the diagnostic accuracy of the PMcardio compared with the interpretation of the expert panel for the outcomes of interest using the Android platform. As a secondary analysis we presented Cohen’s kappa and 95% CI for agreement between Android vs iPhone for the outcomes of interest in order to assess whether the application can be used equally in clinical practice by users of both platforms.^13^

To assess whether the indication for ECG, ECG quality, or ECG format could be of influence on PMcardio diagnostic accuracy, we provided subgroup analyses. We validated PMcardio for the primary and key secondary outcomes in the subsets of patients who presented with cardiac symptoms; those with excellent ECG quality, defined as no to mild overall noise (mild noise was defined as presence of baseline irregularities but with P wave still discernible) and up to 1 out of 12 leads with baseline drift in the 12-lead ECG; and those with the most frequently used ECG configurations (3 × 4 leads + 1 RS, n = 202; and 6 × 2 leads + 1 RS, n = 71; see Supplemental Figure 1 for examples of these ECG formats). We also provided a sensitivity analysis where only PMcardio ECG assessments with high level of confidence, not low or mid level of confidence, were counted as positive for the index test, with the aim of assessing the accuracy of the confidence levels provided by PMcardio. Finally, we provided a comparison between PMcardio (as assessed on Android platform) and the 12-lead ECG device’s in-built automated interpretation algorithm (AIA) in those with available 12-lead ECG AIA results (n = 45) in order to compare PMcardio’s interpretation with that of commonly available automated ECG interpretation algorithm.

We displayed descriptives of discrete variables as number and percentages and of continuous variables as median and interquartile range. We compared continuous variables using Student *t* test in case of normally distributed data or Mann–Whitney *U* test in case of non-normally distributed data, and proportions using the Fisher exact test or Pearson χ^2^ test. We assessed normality of distribution of continuous data using the Q-Q plot and Kolmogorov–Smirnov test. We used 2-tailed tests. We evaluated statistical significance in all analyses at the .05 level. We performed our analyses using R version 4.0.3^14^ with the dplyr, expss, haven, table1, and vcd packages; SPSS version 28.0.1.1,^15^ and MedCalc version 20.118.^16^

**Table 1:** Baseline characteristics

	All	Excellent ECG quality sample†	*P* value
	(n = 290)	(n = 189)	
Age (years)	67.0 (55.3–74.0)	67.0 (56.0–75.0)	.925
Female	138 (47.6)	85 (45.0)	.273
Reason for ECG‡			
New symptoms	173 (59.7)	105 (55.6)	.069
Cardiovascular risk management	31 (10.7)	23 (12.2)	.360
Known diabetes mellitus	47 (16.2)	34 (18.0)	.337
Known ischemic heart disease	14 (4.8)	13 (6.9)	.052
Known heart rhythm disorder	6 (2.1)	4 (2.1)	1.000
Other	19 (6.6)	10 (5.3)	.348
Symptoms§			
Palpitations	74 (25.5)	39 (20.6)	.014
Chest pain	64 (22.1)	44 (23.3)	.595
Dyspnea	37 (12.8)	24 (12.7)	1.000
Light-headedness	23 (7.9)	12 (6.3)	.256
Fatigue	26 (9.0)	17 (9.0)	1.000
(Near) collapse	12 (4.1)	6 (3.2)	.414
Other	31 (10.7)	18 (9.5)	.497
Hypertension	123 (42.4)	79 (41.8)	.741
Heart failure	12 (4.1)	7 (3.7)	.843
Diabetes mellitus	85 (29.3)	60 (31.7)	.339
Prior stroke/TIA	18 (6.2)	13 (6.9)	.694
Atrial fibrillation	30 (10.3)	23 (12.2)	.233
Other arrhythmia	13 (4.5)	9 (4.8)	.987
Valvular disease	13 (4.5)	10 (5.3)	.540
Hypercholesterolemia	68 (23.4)	44 (23.3)	1.000
Peripheral vascular disease	22 (7.6)	17 (9.0)	.314
Coronary heart disease	30 (10.3)	20 (10.6)	1.000
COPD	30 (10.3)	16 (8.5)	.278
Chronic kidney disease	30 (10.3)	18 (9.5)	.315
Beta-blocker use	57 (19.7)	37 (19.6)	1.000
Sodium channel blocker use	4 (1.4)	2 (1.1)	.910
Potassium channel blocker use	2 (0.7)	2 (1.1)	.770
Calcium channel blocker use	43 (14.8)	28 (14.8)	1.000
Digoxin use	2 (0.7)	2 (1.1)	.770

Data are number (percentage) or median (interquartile range). *P* value is for difference between excellent (n = 189) and non-excellent (n = 101) ECG quality sample.

COPD = chronic obstructive pulmonary disease; ECG = electrocardiogram; IQR = interquartile range; TIA = transient ischemic attack.

† Excellent ECG quality was defined as no or mild noise and/or up to 1 lead with baseline drift.

‡ Patients were assessed as having 1 (primary) reason for ECG.

§ Patients could report multiple symptoms at baseline, and symptoms were not mutually exclusive.

## Results

**Figure 2:**
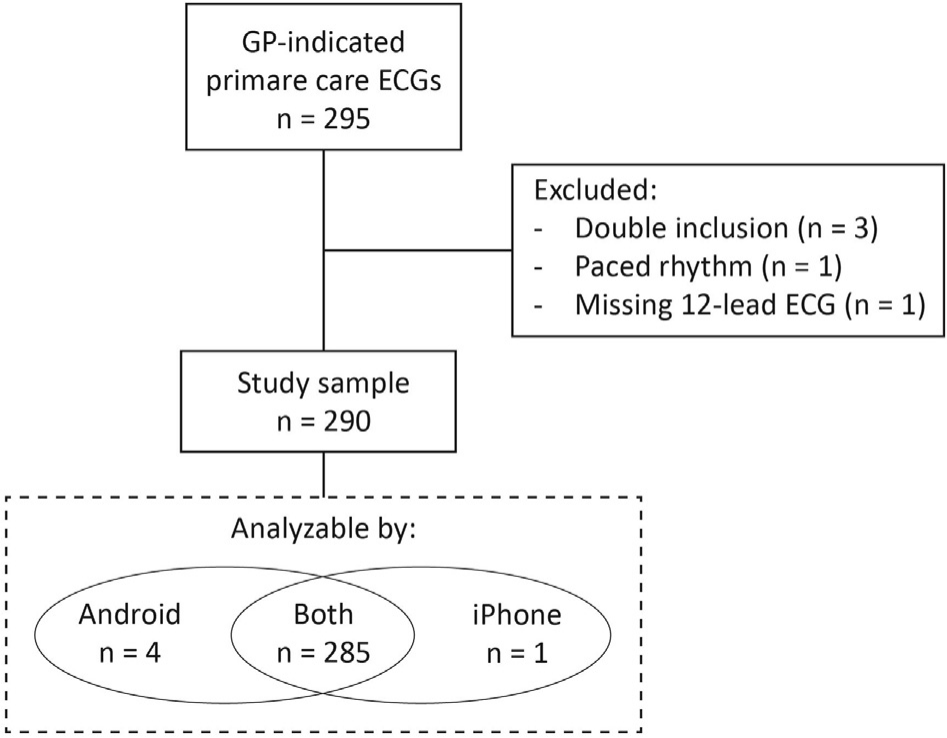
Study flowchart. ECG = electrocardiogram; GP = general practitioner.

The study population consisted of 290 patients, representing a consecutive series of patients who had a 12-lead ECG performed in one of the 11 participating general practices (see Figure 2 for flowchart and reasons for exclusion). Baseline characteristics of included patients are listed in Table 1. Median age was 67 years, 48% were female, and 173 (60%) of patients presented with new symptoms. The majority of these symptoms were either palpitations (26%) or chest pain (22%). The most common comorbidities were hypertension (42%), diabetes mellitus (29%), and hypercholesterolemia (23%).

Of the 290 ECGs, the PMcardio could analyze and produce a diagnostic result in 285 (98%) on both smartphones; with 289 analyzable on the Android device and 286 on the iOS device (Figure 2). The quality of ECGs was judged as excellent by the readers in 189 cases (65%). A list of ECG findings by the expert panel can be found in Table 2. Major ECG abnormalities were found in 71 patients (25%), with AF present in 12%, signs of past or present ischemia present in 9%, and clinically relevant impulse or conduction abnormalities in 9%.

**Table 2:** ECG findings

ECG finding	All (n = 290)	Excellent ECG quality sample† (n = 189)	*P* value
Sinus rhythm	246 (84.8)	160 (84.7)	1.000
Atrial fibrillation or flutter	35 (12.1)	24 (12.7)	.794
Narrow or broad complex tachycardia	5 (1.7)	3 (1.6)	1.000
Atrial junctional rhythm	1 (0.3)	0 (0)	.750
Ectopy	28 (9.7)	22 (11.6)	.175
Bundle branch block	25 (8.6)	19 (10.1)	.332
Left bundle branch block	12 (4.1)	7 (3.7)	.843
Right bundle branch block	5 (1.7)	4 (2.1)	.819
Bifascicular block	7 (2.4)	7 (3.7)	.120
Trifascicular block	1 (0.3)	1 (0.5)	1.000
AV block	13 (4.5)	6 (3.2)	.240
High-degree AV block	0 (0)	0 (0)	1.000
Prolonged QT interval (QTc >480 ms)	5 (1.7)	4 (2.1)	.819
Left ventricular hypertrophy	11 (3.8)	9 (4.8)	.390
Significant ST elevation	6 (2.1)	2 (1.1)	.222
Significant ST depression	6 (2.1)	5 (2.6)	.610
T-wave inversion	9 (3.1)	6 (3.2)	1.000
Pathologic Q waves	8 (2.8)	7 (3.7)	.333
Major ECG abnormalities	71 (24.5)	51 (27.0)	.226
Indication of (past) ischemia	26 (9.0)	18 (9.5)	.811
Clinically relevant impulse or conduction abnormality	27 (9.3)	19 (10.1)	.702

Data are number (percentage). *P* value is for difference between excellent (n = 189) and non-excellent (n = 101) ECG quality sample.

AV = atrioventricular; ECG = electrocardiogram; QTc = corrected QT interval; ST = ST interval.

† Excellent ECG quality was defined as no or mild noise and/or up to 1 lead with baseline drift.

### Diagnostic accuracy of the PMcardio in the overall sample

Data on diagnostic accuracy with calculated 95% CIs for the primary and key secondary outcomes in the overall sample are summarized in Table 3. The PMcardio app had a sensitivity and specificity for major ECG abnormalities of 86% (95% CI: 76%–93%) and 92% (95% CI: 87%–95%), respectively. The corresponding PPV and NPV were 77% (95% CI: 68%–84%) and 95% (95% CI: 92%–97%), respectively. For AF, sensitivity and specificity were high at 97% (95% CI: 85%–100%) and 99% (95% CI: 97%–100%), respectively, with PPV and NPV of 94% (95% CI: 81%–99%) and 100% (95% CI: 97%–100%), respectively. For ECG signs of past or present ischemia the PMcardio performed suboptimally, with a sensitivity of 54% (95% CI: 33%–73%) and a specificity of 96% (95% CI: 93%–98%) and a PPV and NPV of 58% (95% CI: 41%–74%) and 96% (95% CI: 93%–97%), respectively. The sensitivity, specificity, PPV, and NPV of clinically relevant impulse or conduction abnormalities were 89% (95% CI: 71%–98%), 92% (95% CI: 89%–95%), 55% (95% CI: 44%–65%), and 99% (95% CI: 97%–100%), respectively. The performance of the PMcardio application for the primary and key secondary outcomes was comparable between the Android and iOS platforms, with a very high level of agreement (kappa exceeding 0.90 in all analyses).

**Table 3:** Validation of the PMcardio app for the primary and key secondary outcomes in the overall sample (n = 290)

Major ECG abnormalities										
		Ref +	Ref -	Sensitivity	Specificity	LR+	LR-	PPV	NPV	Kappa†
PMcardio	+	61	18	85.9% (75.6–93.0)	91.7% (87.3–95.0)	10.4 (6.6–16.4)	0.15 (0.09–0.27)	77.2% (68.3–84.2)	95.2% (91.8–97.3)	0.95 (0.91–0.99)
	-	10	200							

Atrial fibrillation or flutter										
		Ref +	Ref -	Sensitivity	Specificity	LR+	LR-	PPV	NPV	Kappa†
PMcardio	+	34	2	97.1% (85.1–99.9)	99.2% (97.2–99.9)	123.4 (31.0–491.2)	0.03 (0.00–0.20)	94.4% (81.0–98.5)	99.6% (97.3–99.9)	1.00 (1.00–1.00)
	-	1	252							

Indication of (past) ischemia										
		Ref +	Ref -	Sensitivity	Specificity	LR+	LR-	PPV	NPV	Kappa†
PMcardio	+	14	10	53.6% (33.4–73.4)	96.2% (93.1–98.2)	14.2 (7.0–28.6)	0.48 (0.32–0.73)	58.3% (40.9–73.9)	95.5% (93.3–97.0)	0.91 (0.81–1.00)
	-	12	253							

Clinically relevant impulse or conduction abnormality										
		Ref +	Ref -	Sensitivity	Specificity	LR+	LR-	PPV	NPV	Kappa†
PMcardio	+	24	20	88.9% (70.8–97.7)	92.4% (88.5–95.3)	11.6 (7.5–18.1)	0.12 (0.04–0.35)	54.6% (43.6–65.1)	98.8% (96.5–99.6)	0.91 (0.84–0.98)
	-	3	242							

Data are point estimate (95% confidence interval). Reference in each analysis is expert panel consensus on presence of the outcome of interest.

ECG = electrocardiogram; LR+ = positive likelihood ratio; LR- = negative likelihood ratio; NPV = negative predictive value; PPV = positive predictive value.

† Kappa for interobserver agreement between Android and iPhone.

Diagnostic accuracy of the PMcardio for the secondary outcomes is shown in Table 4. Sensitivity was highest for any BBB, at 92% (95% CI: 74%–99%), and lowest for LVH, at 64% (95% CI: 31%–89%). Specificity was over 80% for all secondary outcomes, again with best performance for any BBB at 97% (95% CI: 94%–99%). Kappa for agreement between Android and iOS ranged from 0.83 to 0.93 for the secondary outcomes.

**Table 4:** Validation of the PMcardio app for secondary outcomes in the overall sample (n = 290)

Significant ST deviation										
		Ref +	Ref -	Sensitivity	Specificity	LR+	LR-	PPV	NPV	Kappa†
PMcardio	+	8	16	66.7% (34.9–90.1)	94.2% (90.8–96.7)	11.5 (6.2–21.5)	0.35 (0.16–0.79)	33.3% (21.2–48.2)	98.5% (96.7–99.3)	0.91 (0.81–1.00)
	-	4	261							

Left ventricular hypertrophy										
		Ref +	Ref -	Sensitivity	Specificity	LR+	LR-	PPV	NPV	Kappa†
PMcardio	+	7	42	63.6% (30.7–89.1)	84.9% (80.1–88.9)	4.2 (2.5–7.1)	0.43 (0.2–0.9)	14.3% (9.0–22.0)	98.3% (96.4–99.2)	0.83 (0.74–0.91)
	-	4	236							

Ectopy										
		Ref +	Ref -	Sensitivity	Specificity	LR+	LR-	PPV	NPV	Kappa†
PMcardio	+	25	30	89.3% (71.8–97.7)	88.5% (84.0–92.1)	7.8 (5.4–11.1)	0.12 (0.04–0.35)	45.5% (36.8–54.4)	98.7% (96.4–99.6)	0.86 (0.79–0.94)
	-	3	231							

Any BBB										
		Ref +	Ref -	Sensitivity	Specificity	LR+	LR-	PPV	NPV	Kappa†
PMcardio	+	23	8	92.0% (74.0–99.0)	97.0% (94.1–98.7)	30.4 (15.2–60.7)	0.08 (0.02–0.31)	74.2% (59.0–85.2)	99.2% (97.1–99.8)	0.93 (0.85–1.00)
	**-**	2	256							

Data are point estimate (95% confidence interval). Reference in each analysis is expert panel consensus on presence of the outcome of interest.

BBB = bundle branch block; LR+ = positive likelihood ratio; LR- = negative likelihood ratio; NPV = negative predictive value; PPV = positive predictive value.

† Kappa for interobserver agreement between Android and iPhone.

### Additional analyses

Diagnostic accuracy for the primary and key secondary outcomes in the subgroup of patients presenting for new symptoms is shown in Supplemental Table 3. PMcardio’s sensitivity for ECG indications for (past) myocardial ischemia was remarkably higher in this subgroup compared with the overall sample, at 80% (95% CI: 52%–96%). Diagnostic accuracy for the other outcomes was similar to that in the overall analysis.

Supplemental Table 4 depicts the findings within the subgroup of ECGs of excellent quality. Overall the diagnostic accuracy of PMcardio was comparable with the main analysis, except for clinically relevant impulse or conduction abnormalities, for which sensitivity improved to 100% (95% CI: 82%–100%), with comparable specificity (94%; 95% CI: 90%–97%), resulting in an improved PPV of 66% (51%–78%) and perfect NPV of 100%.

Validation of PMcardio in ECGs with 3 × 4 leads + 1 RS and 6 × 2 leads + 1 RS formats resulted in comparably high diagnostic accuracy for all analyzed outcomes except for indication of (past) ischemia (Supplemental Tables 5 and 6, respectively). For this outcome, sensitivity among ECGs with 3 × 4 leads + 1 RS was low at 33% (95% CI: 12%–62%), while sensitivity for this outcomes among ECGs with a 6 × 2 leads + 1 RS format was 89% (95% CI: 52%–100%). Whether this finding could be explained by the longer duration per lead of the 6 × 2 leads + 1 RS format (4.5 vs 2.5 seconds; see Supplemental Figure 1) was not certain from our data.

When scoring only high-confidence-level ECG assessment as positive, PMcardio generally showed improved specificity and LR+, but remarkably reduced sensitivity (Supplemental Table 7). As in the main analysis, lowest accuracy was seen for the indication of (past) ischemia key secondary outcome, with sensitivity 27% (95% CI: 12%–48%), and kappa 0.61 (95% CI: 0.29-0.92) for agreement between the Android and iOS platforms. Among the secondary outcomes, marked improvement was seen in assessing presence of ectopy when counting only high-confidence-level reference test results as positive (Supplemental Table 8). However, raising the threshold for a positive test to high confidence level was considerably less accurate for diagnosing significant ST elevation, as shown by sensitivity of 17% (95% CI: 2%–48%) for Android with kappa 0.61 for this outcome.

In the subset of ECGs with available 12-lead ECG AIA results, PMcardio’s interpretation showed a trend toward better performance vs reference ECG than the 12-lead ECG’s AIA for the primary and key secondary outcomes (Supplemental Table 9). Agreement as assessed by the kappa estimate and 95% CI was low in all analyses except for that on AF. The analyses were limited in power owing to the small sample size.

## Discussion

To our knowledge this is the first study to independently validate the PMcardio, a promising digital diagnostic assistant for physicians. Overall we found that the diagnostic properties of the PMcardio smartphone application performed well against our reference standard, a panel of expert ECG readers. The diagnostic tool was particularly reliable in diagnosing AF. We found no differences in the diagnostic performance between 2 major smartphone platforms (iOS and Android). We generally saw consistent reliability irrespective of ECG quality, except for impulse or conduction abnormalities, which were more often correctly diagnosed in ECGs of higher quality. PMcardio’s performance in detecting indications of (past) myocardial ischemia was less robust in our overall, low-risk sample of older primary care patients presenting for ECG for any indication, with poorer sensitivity regardless of ECG quality. However, there were indications that diagnostic accuracy for ischemic markers was better in patients presenting for new symptoms, and in ECGs with 6 × 2 leads + 1 RS format.

### Strengths and limitations

Our study has a number of strengths. First, we included consecutive patients who underwent 12-lead ECG as part of routine medical practice, resulting in a representative sample of Dutch primary care patients who undergo ECG for any indication. Second, we ensured standardized interpretation of all recordings by blinded ECG assessment in random order. Third, we tested the PMcardio smartphone application separately on 2 major smartphone software platforms. The high inter-platform agreement shown in our study was expected given that the only difference between these platforms should be the camera resolution and subsequent digitization of the ECG, after which the ECG analysis is centrally performed. Still, including these results can be important in showing potential users that the application can be employed equally by owners of both Android and iOS smartphones. Fourth, we performed additional analyses to assess which factors influenced the smartphone application’s diagnostic performance. Finally, though limited by sample size, we offered a comparison of PMcardio’s automated ECG interpretation with that of standard automated interpretation by the 12-lead ECG device.

Several limitations deserve to be mentioned. Our validation analyses were limited to the ECG assessment module and did not validate PMcardio’s clinical and diagnostic recommendations module. The current study was therefore not designed to determine the added value of the PMcardio smartphone application when provided to GPs in routine care, but rather was designed to describe the test characteristics of assessing the presence or absence of ECG abnormalities in routine primary care 12-lead ECGs. As such, the current study was not designed to study whether the availability of a smartphone application for automated ECG assessment would change ECG use, diagnosis, or patient management. Given that PMcardio’s ECG assessment module is solely based on the processing of the 12-lead ECG signal, not providing the app with clinical information for each analyzed case should not have been a limitation. Furthermore, the sample size in our study, though comparable to previous validation studies validating novel techniques in primary care ECG samples, was limited.^9^ Subgroup analyses were naturally further affected by this limitation but were deemed sufficiently deserving of investigation to be included in the report. As indicated in Supplemental Table 2, the outcomes as reported by PMcardio did not always completely match those that we deemed the clinically most relevant outcomes for primary care decision-making (eg, our “LVH” vs PMcardio’s broader “ventricular hypertrophy”), potentially explaining reduced accuracy found for such outcomes. In comparing the PMcardio application to standard 12-lead AIA, the analyses were limited by further reduced sample size. Moreover, there was heterogeneity of employed 12-lead ECG devices used by participating practices, as well as missing data on the model and make of each practice’s ECG device, hindering a direct comparison to 1 particular 12-lead ECG device and its AIA. Finally, while the patient sample was sufficient for our primary and key secondary ECG abnormalities of interest, the prevalence of individually studied outcomes was rather low, which limited our ability to perform further subgroup analyses and explains the relatively wide confidence intervals in analyses on individual ECG outcomes.

### Clinical relevance

Adequate ECG interpretation is paramount to make adequate referral and/or treatment decisions, which means that GPs must have competence in ECG interpretation. Unfortunately the ECG interpretation skill of GPs is not on par with that of cardiologists,^17^, ^18^, ^19^, ^20^, ^21^ with previous work indicating that GPs incorrectly assess 1 in 5 normal ECGs as abnormal.^22^ We saw that PMcardio was especially reliable in assessing presence or absence of AF. Given the mixed evidence on GPs’ ability to assess AF on ECG signal,^17^^,^^20^, ^21^, ^22^ PMcardio could be of particular use for AF diagnosis as well as AF management when combined with PMcardio’s guideline-directed treatment recommendation module.

Multiple studies have suggested that the discrepancy between GPs and cardiologists is especially high in assessing ECGs for ischemic markers.^20^, ^21^, ^22^ When combined with the often (semi)acute nature of questions on the presence or absence of ischemic markers on 12-lead ECG, a tool that reliably assists GPs in this assessment could be highly clinically relevant. We note that some primary care guidelines discourage the use of 12-lead ECG in assessment of patients with an acute coronary syndrome suspicion on the grounds that a singular ECG has insufficient NPV compared to serum biomarkers of myocardial ischemia.^23^ However, in settings where such measurements are unavailable, or where the threshold for cardiologic consultation is high, a tool to assist GPs in establishing ischemic markers on 12-lead ECG is all the more relevant.

Our data suggest that PMcardio has difficulties in correctly assessing signs of (past) ischemia on 12-lead ECG. It is important to note, however, that the low sensitivity for this composite key secondary outcome was mainly due to the algorithm often not acknowledging pathologic Q waves, while accuracy for the more acute secondary outcome “significant ST deviations” (not including Q waves or T-wave inversion) was more favorable. Though it is often a nonacute finding, we included Q waves in the definition of our composite outcome, as Q waves could be relevant for GPs in assessing whether a patient with, for example, suspicion of heart failure should be referred for further cardiologic work-up owing to signs of past myocardial ischemia.^24^ As seen by the higher diagnostic accuracy for ST deviations, especially in the subset of symptomatic patients, PMcardio seems better equipped for more acute cases than those in our overall sample of consecutive, older primary care patients undergoing ECG for any indication. A possible explanation is that the PMcardio algorithm was trained in a higher-prevalence ECG dataset compared to the sample in which it was currently validated.^25^ The main lesson for our intended audience—GPs who wish to assess whether PMcardio could be a useful addition to their 12-lead ECG interpretation—on the use of PMcardio for assessing signs of ischemia seems to be that the current version of the application seems to be of best use for signs of (semi)acute ischemia in symptomatic patients. Given that our work, however, is a first validation attempt with considerable uncertainties, the question to what extent PMcardio can provide more efficient ECG interpretation as well as treatment recommendation in (semi)acute symptomatic primary care patients warrants further investigation.

Tools to help improve ECG interpretation include the use of interpretation software and the possibility of (digital) consultation of a cardiologist. Current interpretation software is not reliable enough to obviate physician over-reading and confirmation.^6^^,^^26^ However, even in this scenario adequate ECG interpretation remains questionable, with some studies suggesting an increase in sensitivity when combining GP interpretation with automatic algorithm software, while others do not.^17^^,^^18^ The alternative is a (digital) cardiologist consultation service. This option is more reliable, but is more time consuming and costly. It does not work well with the flow of care, in which a (rapid) response may be required. With this in mind, the PMcardio provides an easy-to-use solution for physicians who have to interpret an ECG but lack the routine ECG interpretation skills. As such, the PMcardio may offer a welcome point-of-care diagnostic aid in primary care, particularly when it comes to ruling out cardiac arrhythmias.

### Prior work

Computerized interpretation of ECGs has been around for several decades and was introduced to improve the correct interpretation, thereby facilitating correct decision-making and reducing harm and costs.^26^ Despite improvements, the diagnostic accuracy of these computerized interpretations remains limited, with particular concern for false-positives.^26^ This may inflict potential harm owing to unnecessary diagnostic investigations and interventions—which is not a theoretical risk, as around the world millions of ECGs are recorded annually in which automatic interpretation is reviewed by a clinician with relatively little ECG experience.^26^ On the upside, when the ECG software algorithms determine the ECG to be normal, data from multiple settings indicate that one can safely conclude that this is correct.^27^

Taking automatic 12-lead ECG interpretations to the physician’s smartphone is a new frontier that provides a number of new opportunities. The premise of PMcardio was to deliver an in-your-pocket clinical assistant that uses ECG interpretation as a starting point for providing evidence-based diagnostic and/or treatment recommendations. However, the scientific body of evidence thus far is limited. The diagnostic performance of the PMcardio was previously assessed by the inventors and company who have developed the application.^25^ In their benchmark report they describe to have tested the underlying algorithms on more than 12,000 cases for 38 ECG abnormalities. The report lists similar sensitivity and specificity compared to our work for AF (96% and 100%, respectively) and BBB (95% and 99%, respectively), but higher sensitivity for suspected ST-elevation and non-ST-elevation myocardial infarction (99% and 83%, respectively). Specificity for the latter outcomes (92% and 98%, respectively) was similar to that in our work.

The manufacturers also compared PMcardio (version 2.5) to the individual assessment of GPs and found that the application was more reliable for heart blocks, infarctions, ectopies, hypertrophies, arrhythmias, and axis deviations. Moreover, they found the application even to surpass individual cardiologist assessments for heart blocks, infarctions, and axis deviations.^25^ In our study we did not perform a head-to-head comparison with clinicians (either cardiologists or non-cardiologists), as we deemed it more relevant to evaluate how the smartphone application would compare as a stand-alone tool compared with an expert panel of ECG readers, which we consider to be the gold standard.

### Future work

Further study is required to evaluate the safety and efficacy of the PMcardio in the hands of GPs, in terms of both diagnostic accuracy and improved care and, ultimately, clinical outcomes. This is especially relevant given PMcardio’s modest results in detecting indications for (past) myocardial ischemia in our overall sample. An important study in that regard is the PMCARDIO-PT1, which is a multicenter randomized clinical trial, aimed to enroll 836 patients of at least 55 years of age to study whether the use of the PMcardio clinical assistant results in a more efficient patient management in primary care and more accessible specialized care compared to usual standards of care.^28^ Additionally, it also aims to assess time savings and cost-saving implications of increased availability of specialized care at the primary care level. The PMCARDIO-PT1 study is especially relevant because it will include only patients with cardiovascular symptoms, given that our results indicate a trend toward better performance for diagnosing ECG signs of (past) myocardial ischemia in patients who presented for new symptoms vs in patients free of symptoms undergoing protocolized ECG. In addition to this study, it would be worthwhile to evaluate how this application performs in an urgent or out-of-hours primary care setting, where the need for a rapid and reliable ECG interpretation and triage tool is of particular relevance, while immediate cardiologic consultancy may not be immediately available. There is thus still enough ground to cover. Is the PMcardio a “cardiologist in your pocket”? No, it is not (yet)—but it is getting close.

## Conclusion

A smartphone application developed to interpret 12-lead ECGs was found to have good overall diagnostic accuracy in a primary care setting, and near-perfect properties for diagnosing AF when compared with a panel of expert readers. However, caution is warranted when assessing ECGs for signs of (past) myocardial ischemia in asymptomatic patients, as well as for impulse or conduction abnormalities in ECGs of suboptimal quality. Our study provides important insights for GPs who are in need of a point-of-care ECG interpretation assistant, who are in doubt of their own interpretation skills, and in whom consulting a cardiologist presents a logistical or temporal threshold.
