# Supplementary material for: Diagnostic accuracy of the PMcardio smartphone application for artificial intelligence–based interpretation of electrocardiograms in primary care (AMSTELHEART-1)
Source: Cardiovasc Digit Health J. 2023 Apr 5;4(3):80–90. doi: 10.1016/j.cvdhj.2023.03.002 (PMC10282008; doi:10.1016/j.cvdhj.2023.03.002)
Supplement: Supplementary Material [file mmc1.docx]

**Supplement to Himmelreich, J.C.L. and Harskamp, R.E. “Diagnostic accuracy of the PMcardio smartphone application for artificial intelligence-based interpretation of electrocardiograms in primary care (AMSTELHEART-1)”**

**Supplementary Figure S1.** Comparison of the 3x4 + 1 rhythm strip and 6x2 + 1 rhythm strip ECG formats

**
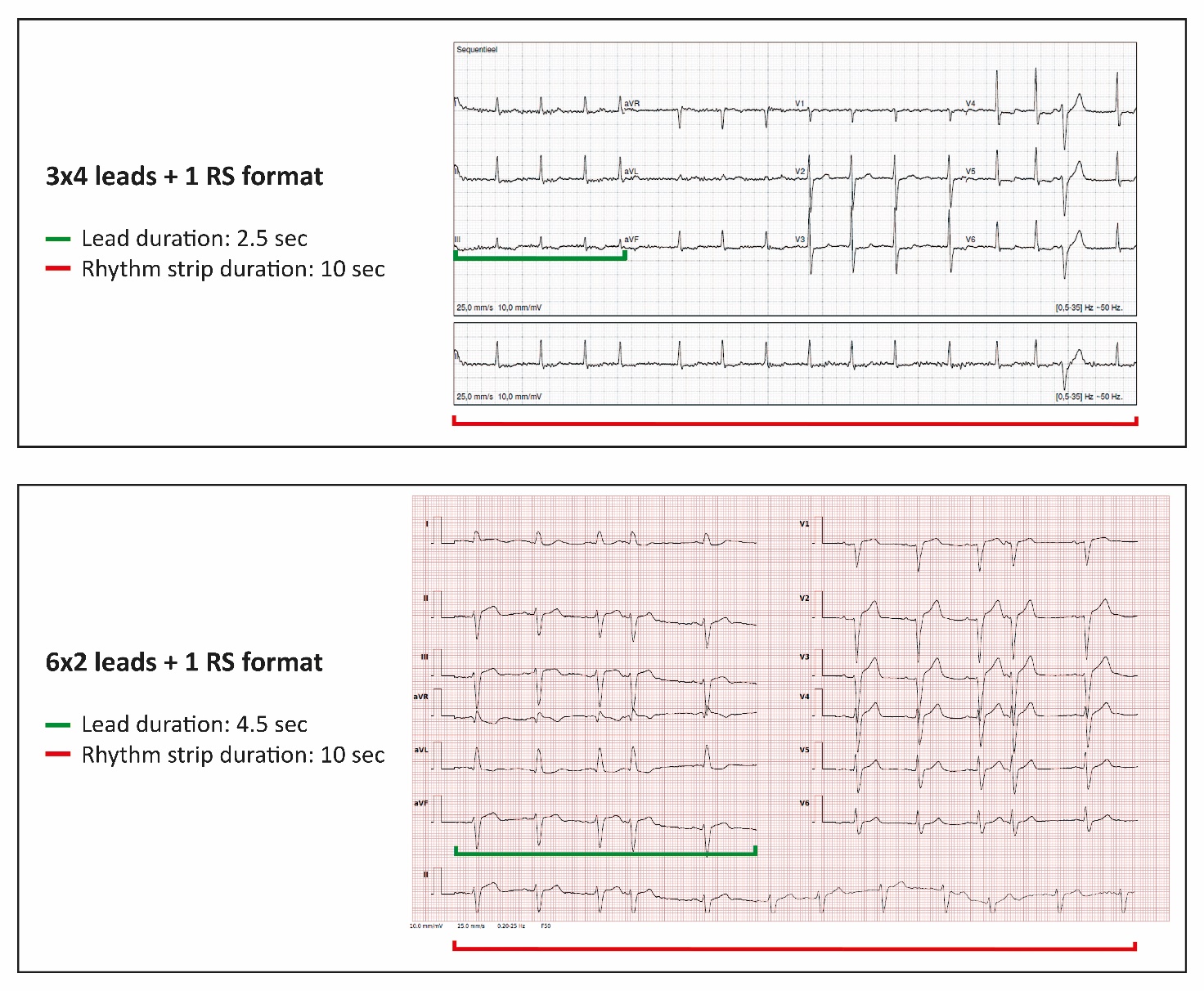
**

RS, rhythm strip; sec, seconds.

**Supplementary Table S1.** PMcardio ECG categories

| **Category** | **ECG entity** |
| --- | --- |
| **Heart blocks** | 1^st^ degree AV block  2^nd^ degree AV block, type Wenckebach  Higher degree AV block  RBBB  LBBB  LAFB  LPFB  Nonspecific intraventricular conduction delay  Bifascicular block (RBBB + LAFB)  Bifascicular block (RBBB + LPFB)  Trifascicular block (RBBB + LAFB + 1^st^ degree AV block)  Trifascicular block (RBBB + LPFB + 1^st^ degree AV block)  Incomplete RBBB  Incomplete LBBB |
| **Infarctions** | Suspected ST-elevation ACS  Suspected NonST-elevation ACS |
| **Ectopies** | Premature complexes |
| **Hypertrophies** | Suspected atrial enlargement  Suspected ventricular hypertrophy |
| **Rhythms** | Sinus rhythm  Sinus bradycardia  Sinus tachycardia  Paced rhythm |
| **Arrhythmias** | Atrial fibrillation  Atrial flutter  Supraventricular tachycardia  Suspected junctional rhythm  Wide QRS tachycardia  Wide QRS rhythm  Atrial fibrillation with rapid ventricular response  Atrial fibrillation with slow ventricular response  Atrial flutter with rapid ventricular response  Atrial flutter with slow ventricular response  Suspected junctional bradycardia  Suspected accelerated junctional rhythm  Idioventricular rhythm |
| **Axis** | Normal axis  Left cardiac axis deviation  Right cardiac axis deviation  Extreme cardiac axis deviation |
| **Other** | Suspected long QT syndrome  Suspected short QT syndrome |

ACS, acute coronary syndrome; AV, atrioventricular; LAFB, left anterior fascicular block; LBBB, left bundle branch block; LPFB, left posterior fascicular block; QRS, QRS interval; QTc, corrected QT interval; RBBB, right bundle branch block.

**Supplementary Table S2.** Outcome definitions and corresponding PMcardio ECG categories

|  | **Outcome definition** | **Reference ECG categories in definition** | **PMcardio ECG categories in definition** |
| --- | --- | --- | --- |
| *Primary outcome* | Any major ECG abnormality (MEA; primary outcome) | Atrial fibrillation  Atrial flutter  Pathological Q-waves  ST elevation  ST depression  T wave inversion  High-degree AV block  LBBB  Bifascicular block  Trifascicular block  Prolonged QT interval (QTc >480ms)  Narrow complex tachycardia (QRS≤120ms)  Broad complex tachycardia (QRS>120ms) | Atrial fibrillation  Atrial flutter  Atrial fibrillation with rapid ventricular response  Atrial fibrillation with slow ventricular response  Atrial flutter with rapid ventricular response  Atrial flutter with slow ventricular response  Suspected ST-elevation ACS  Suspected NonST-elevation ACS  Higher degree AV block  LBBB  Bifascicular block (RBBB + LAFB)  Bifascicular block (RBBB + LPFB)  Trifascicular block (RBBB + LAFB + 1^st^ degree AV block)  Trifascicular block (RBBB + LPFB + 1^st^ degree AV block)  Prolonged QTc time  Suspected long QT syndrome  Supraventricular tachycardia  Wide QRS tachycardia |
| *Key secondary outcomes* | Atrial fibrillation or flutter | atrial fibrillation  atrial flutter | Atrial fibrillation  Atrial flutter  Atrial fibrillation with rapid ventricular response  Atrial fibrillation with slow ventricular response  Atrial flutter with rapid ventricular response  Atrial flutter with slow ventricular response |
|  | Indication of (past) ischemia | Pathological Q waves  ST elevation  ST depression  T wave inversion | Suspected ST-elevation ACS  Suspected NonST-elevation ACS |
|  | Clinically relevant impulse or conduction abnormality | Accelerated junctional rhythm  LBBB  RBBB  Bifascicular block  Trifascicular block  High-degree AVB  Prolonged QT interval (QTc >480ms) | Suspected accelerated junctional rhythm  LBBB  RBBB  Bifascicular block (RBBB + LAFB)  Bifascicular block (RBBB + LPFB)  Trifascicular block (RBBB + LAFB + 1^st^ degree AV block)  Trifascicular block (RBBB + LPFB + 1^st^ degree AV block)  High-degree AVB  Suspected long QT syndrome |
| *Secondary outcomes* | Significant ST deviation | ST elevation  ST deviation | Suspected ST-elevation ACS  Suspected NonST-elevation ACS |
|  | LVH | LVH | Suspected ventricular hypertrophy |
|  | Ectopy | Premature atrial complexes  Premature ventricular complexes | Premature complexes |
|  | Any BBB | LBBB  RBBB  Bifascicular block  Trifascicular block | LBBB  RBBB  Bifascicular block (RBBB + LAFB)  Bifascicular block (RBBB + LPFB)  Trifascicular block (RBBB + LAFB + 1^st^ degree AV block)  Trifascicular block (RBBB + LPFB + 1^st^ degree AV block) |

ACS, acute coronary syndrome; AV, atrioventricular; LAFB, left anterior fascicular block; LBBB, left bundle branch block; LVH, left ventricular hypertrophy; LPFB, left posterior fascicular block; QRS, QRS interval; QTc, corrected QT interval; RBBB, right bundle branch block.

**Supplementary Table S3.** Validation of the PMcardio app for the primary and key secondary outcomes in participants who presented with new symptoms (n = 173).

| **Major ECG abnormalities** | | | | | | | | | | |
| --- | --- | --- | --- | --- | --- | --- | --- | --- | --- | --- |
|  | | **Ref +** | **Ref -** | **Sensitivity** | **Specificity** | **LR+** | **LR-** | **PPV** | **NPV** | **Kappa^1^** |
| **PMcardio** | **+** | 44 | 14 | 91.7%  (80.0-97.7) | 88.7%  (81.8-93.7) | 8.1  (4.9-13.4) | 0.09  (0.04-0.24) | 75.9%  (65.6-83.8) | 96.5%  (91.5-98.6) | 0.95  (0.90-1.00) |
|  | **-** | 4 | 110 |  |  |  |  |  |  |  |
| **Atrial fibrillation or flutter** | | | | | | | | | | |
|  | | **Ref +** | **Ref -** | **Sensitivity** | **Specificity** | **LR+** | **LR-** | **PPV** | **NPV** | **Kappa^1^** |
| **PMcardio** | **+** | 25 | 2 | 96.2%  (80.4-99.9) | 98.6%  (95.1-99.8) | 70.2  (17.7-278.6) | 0.04  (0.01-0.27) | 92.6%  (75.9-99.4) | 99.4%  (96.0-98.0) | 1.00  (1.00-1.00) |
|  | **-** | 1 | 144 |  |  |  |  |  |  |  |
| **Indication of (past) ischemia** | | | | | | | | | | |
|  | | **Ref +** | **Ref -** | **Sensitivity** | **Specificity** | **LR+** | **LR-** | **PPV** | **NPV** | **Kappa^1^** |
| **PMcardio** | **+** | 12 | 9 | 80.0%  (51.9-95.7) | 94.3%  (89.4-97.4) | 14.0  (7.1-27.6) | 0.21  (0.08-0.58) | 57.1%  (40.3-72.5) | 98.0%  (94.7-99.3) | 0.91  (0.82-1.00) |
|  | **-** | 3 | 148 |  |  |  |  |  |  |  |
| **Clinically relevant impulse or conduction abnormality** | | | | | | | | | | |
|  | | **Ref +** | **Ref -** | **Sensitivity** | **Specificity** | **LR+** | **LR-** | **PPV** | **NPV** | **Kappa^1^** |
| **PMcardio** | **+** | 15 | 10 | 83.3%  (58.6-96.4) | 93.5%  (88.4-96.8) | 12.8  (6.8-24.2) | 0.18  (0.06-0.50) | 60.0%  (44.3-73.9) | 98.0  (94.5-99.3) | 0.93  (0.85-1.00) |
|  | **-** | 3 | 144 |  |  |  |  |  |  |  |

Data are point estimate (95% confidence interval). Reference in each analysis is expert panel consensus on presence of the outcome of interest.

ECG, electrocardiogram; LR+, positive likelihood ratio; LR-, negative likelihood ratio; NPV, negative predictive value; PPV, positive predictive value.

^1^Kappa for interobserver agreement between Android and iPhone.

**Supplementary Table S4.** Validation of the PMcardio app for the primary and key secondary outcomes in participants with excellent ECG quality (n = 189).

| **Major ECG abnormalities** | | | | | | | | | | |
| --- | --- | --- | --- | --- | --- | --- | --- | --- | --- | --- |
|  | | **Ref +** | **Ref -** | **Sensitivity** | **Specificity** | **LR+** | **LR-** | **PPV** | **NPV** | **Kappa^1^** |
| **PMcardio** | **+** | 43 | 9 | 84.3%  (71.4-93.0) | 93.5%  (88.0-97.0) | 12.9  (6.8-24.6) | 0.17  (0.09-0.32) | 82.7%  (71.5-90.1) | 94.2%  (89.5-96.8) | 0.97  (0.94-1.00) |
|  | **-** | 8 | 129 |  |  |  |  |  |  |  |
| **Atrial fibrillation or flutter** | | | | | | | | | | |
|  | | **Ref +** | **Ref -** | **Sensitivity** | **Specificity** | **LR+** | **LR-** | **PPV** | **NPV** | **Kappa^1^** |
| **PMcardio** | **+** | 23 | 1 | 95.8%  (78.9-99.9) | 99.4%  (96.7-100.0) | 158.1  (22.4-1118) | 0.04  (0.01-0.29) | 95.8%  (76.5-99.4) | 99.4%  (96.0-99.9) | 1.00  (1.00-1.00) |
|  | **-** | 1 | 164 |  |  |  |  |  |  |  |
| **Indication of (past) ischemia** | | | | | | | | | | |
|  | | **Ref +** | **Ref -** | **Sensitivity** | **Specificity** | **LR+** | **LR-** | **PPV** | **NPV** | **Kappa^1^** |
| **PMcardio** | **+** | 9 | 4 | 50.0%  (26.0-74.0) | 97.7%  (94.1-99.4) | 21.4  (7.3-62.5) | 0.51  (0.32-0.81) | 69.2%  (43.5-86.8) | 94.9%  (92.1-96.7) | 0.91  (0.79-1.00) |
|  | **-** | 9 | 167 |  |  |  |  |  |  |  |
| **Clinically relevant impulse or conduction abnormality** | | | | | | | | | | |
|  | | **Ref +** | **Ref -** | **Sensitivity** | **Specificity** | **LR+** | **LR-^2^** | **PPV** | **NPV^3^** | **Kappa^1^** |
| **PMcardio** | **+** | 19 | 10 | 100.0%  (82.4-100.0) | 94.1%  (89.5-97.1) | 17.0  (9.5-31.0) | - | 65.5%  (51.0-77.6) | 100.0% | 0.96  (0.90-1.00) |
|  | **-** | 0 | 160 |  |  |  |  |  |  |  |

Data are point estimate (95% confidence interval). Reference in each analysis is expert panel consensus on presence of the outcome of interest.

ECG, electrocardiogram; LR+, positive likelihood ratio; LR-, negative likelihood ratio; NPV, negative predictive value; PPV, positive predictive value.

^1^Kappa for interobserver agreement between Android and iPhone.

^2^Negative likelihood ratio not calculable in absence of false-negatives.

^3^Negative predictive value 95% confidence interval not calculable in absence of false-negatives

**Supplementary Table S5.** Validation of the PMcardio app for the primary and key secondary outcomes in ECGs with 3x4 leads + 1 rhythm strip format (n = 202).

| **Major ECG abnormalities** | | | | | | | | | | |
| --- | --- | --- | --- | --- | --- | --- | --- | --- | --- | --- |
|  | | **Ref +** | **Ref -** | **Sensitivity** | **Specificity** | **LR+** | **LR-** | **PPV** | **NPV** | **Kappa^1^** |
| **PMcardio** | **+** | 35 | 9 | 77.8%  (62.9-88.8) | 94.2%  (89.3-97.3) | 13.5  (7.2-25.9) | 0.24  (0.14-0.41) | 79.6%  (66.9-88.2) | 93.6%  (89.5-96.2) | 1.00  (1.00-1.00) |
|  | **-** | 10 | 147 |  |  |  |  |  |  |  |
| **Atrial fibrillation or flutter** | | | | | | | | | | |
|  | | **Ref +** | **Ref -** | **Sensitivity** | **Specificity** | **LR+** | **LR-^2^** | **PPV** | **NPV^3^** | **Kappa^1^** |
| **PMcardio** | **+** | 21 | 2 | 100.0%  (83.9-100.0) | 98.9%  (96.0-99.9) | 90.0  (22.7-357.1) | - | 91.3%  (72.6-97.7) | 100.0% | 1.00  (1.00-1.00) |
|  | **-** | 0 | 178 |  |  |  |  |  |  |  |
| **Indication of (past) ischemia** | | | | | | | | | | |
|  | | **Ref +** | **Ref -** | **Sensitivity** | **Specificity** | **LR+** | **LR-** | **PPV** | **NPV** | **Kappa^1^** |
| **PMcardio** | **+** | 5 | 4 | 33.3%  (11.8-61.6) | 97.9%  (94.6-99.4) | 15.5  (4.7-51.7) | 0.68  (0.48-0.98) | 55.6%  (27.3-80.7) | 94.8%  (92.7-96.3) | 0.94  (0.82-1.00) |
|  | **-** | 10 | 182 |  |  |  |  |  |  |  |
| **Clinically relevant impulse or conduction abnormality** | | | | | | | | | | |
|  | | **Ref +** | **Ref -** | **Sensitivity** | **Specificity** | **LR+** | **LR-** | **PPV** | **NPV** | **Kappa^1^** |
| **PMcardio** | **+** | 15 | 13 | 93.8%  (69.8-99.8) | 93.0%  (88.3-96.2) | 13.4  (7.8-22.9) | 0.07  (0.01-0.45) | 53.6%  (40.2-66.4) | 99.4%  (96.3-99.9) | 0.98  (0.94-1.00) |
|  | **-** | 1 | 172 |  |  |  |  |  |  |  |

Data are point estimate (95% confidence interval). Reference in each analysis is expert panel consensus on presence of the outcome of interest.

ECG, electrocardiogram; LR+, positive likelihood ratio; LR-, negative likelihood ratio; NPV, negative predictive value; PPV, positive predictive value.

^1^Kappa for interobserver agreement between Android and iPhone.

^2^Negative likelihood ratio not calculable in absence of false-negatives.

^3^Negative predictive value 95% confidence interval not calculable in absence of false-negatives.

**Supplementary Table S6.** Validation of the PMcardio app for the primary and key secondary outcomes in ECGs with 6x2 leads + 1 rhythm strip format (n = 71).

| **Major ECG abnormalities** | | | | | | | | | | |
| --- | --- | --- | --- | --- | --- | --- | --- | --- | --- | --- |
|  | | **Ref +** | **Ref -** | **Sensitivity** | **Specificity** | **LR+** | **LR-^1^** | **PPV** | **NPV^2^** | **Kappa^3^** |
| **PMcardio** | **+** | 22 | 7 | 100.0%  (84.6-100.0) | 85.7%  (72.8-94.1) | 7.0  (3.5-13.9) | - | 75.9%  (61.3-86.2) | 100% | 0.86  (0.73-0.98) |
|  | **-** | 0 | 42 |  |  |  |  |  |  |  |
| **Atrial fibrillation or flutter** | | | | | | | | | | |
|  | | **Ref +** | **Ref -** | **Sensitivity** | **Specificity** | **LR+^4^** | **LR-** | **PPV^5^** | **NPV** | **Kappa^3^** |
| **PMcardio** | **+** | 10 | 0 | 90.9%  (58.7-99.8) | 100.0%  (94.0-100.0) | - | 0.09  (0.01-0.59) | 100% | 98.4%  (90.3-99.7) | 1.00  (1.00-1.00) |
|  | **-** | 1 | 60 |  |  |  |  |  |  |  |
| **Indication of (past) ischemia** | | | | | | | | | | |
|  | | **Ref +** | **Ref -** | **Sensitivity** | **Specificity** | **LR+** | **LR-** | **PPV** | **NPV** | **Kappa^3^** |
| **PMcardio** | **+** | 8 | 5 | 88.9%  (51.8-99.7) | 91.9%  (82.2-97.3) | 11.0  (4.6-26.4) | 0.12  (0.02-0.77) | 61.5%  (40.1-79.3) | 98.3%  (90.0-99.7) | 0.95  (0.86-1.00) |
|  | **-** | 1 | 57 |  |  |  |  |  |  |  |
| **Clinically relevant impulse or conduction abnormality** | | | | | | | | | | |
|  | | **Ref +** | **Ref -** | **Sensitivity** | **Specificity** | **LR+** | **LR-** | **PPV** | **NPV** | **Kappa^3^** |
| **PMcardio** | **+** | 9 | 5 | 81.8%  (48.2-97.7) | 91.7%  (81.6-97.2) | 9.8  (4.1-23.8) | 0.20  (0.06-0.70) | 64.3%  (42.6-81.3) | 96.5%  (88.7-99.0) | 0.78  (0.60-0.97) |
|  | **-** | 2 | 55 |  |  |  |  |  |  |  |

Data are point estimate (95% confidence interval). Reference in each analysis is expert panel consensus on presence of the outcome of interest.

ECG, electrocardiogram; LR+, positive likelihood ratio; LR-, negative likelihood ratio; NPV, negative predictive value; PPV, positive predictive value.

^1^Negative likelihood ratio not calculable in absence of false-negatives.

^2^Negative predictive value 95% confidence interval not calculable in absence of false-negatives.

^3^Kappa for interobserver agreement between Android and iPhone.

^4^Positive likelihood ratio not calculable in absence of false-positives.

^5^Positive predictive value 95% confidence interval not calculable in absence of false-positives.

**Supplementary Table S7.** Validation of the PMcardio app for the primary and key secondary outcomes in the overall sample (n = 290), assessing only PMcardio assessment with high confidence level as positive for the index test.

| **Major ECG abnormalities** | | | | | | | | | | |
| --- | --- | --- | --- | --- | --- | --- | --- | --- | --- | --- |
|  | | **Ref +** | **Ref -** | **Sensitivity** | **Specificity** | **LR+** | **LR-** | **PPV** | **NPV** | **Kappa^1^** |
| **PMcardio** | **+** | 46 | 4 | 64.8%  (52.5-75.8) | 98.2%  (95.4-99.5) | 35.3  (13.2-94.7) | 0.36  (0.26-0.49) | 92.0%  (81.1-96.9) | 89.5%  (86.2-92.2) | 0.93  (0.87-0.98) |
|  | **-** | 25 | 214 |  |  |  |  |  |  |  |
| **Atrial fibrillation or flutter** | | | | | | | | | | |
|  | | **Ref +** | **Ref -** | **Sensitivity** | **Specificity** | **LR+** | **LR-** | **PPV** | **NPV** | **Kappa^1^** |
| **PMcardio** | **+** | 25 | 1 | 71.4%  (53.7-85.4) | 99.6%  (97.8-100.0) | 181.4  (25.4-1297.5) | 0.29  (0.17-0.48) | 96.2%  (77.8-99.4) | 96.2%  (93.7-97.7) | 0.96  (0.90-1.00) |
|  | **-** | 10 | 253 |  |  |  |  |  |  |  |
| **Indication of (past) ischemia** | | | | | | | | | | |
|  | | **Ref +** | **Ref -** | **Sensitivity** | **Specificity** | **LR+** | **LR-** | **PPV** | **NPV** | **Kappa^1^** |
| **PMcardio** | **+** | 7 | 1 | 26.9%  (11.6-47.8) | 99.6%  (97.9-99.9) | 70.8  (9.1-553.5) | 0.73  (0.58-0.93) | 87.5%  (47.3-98.2) | 93.2%  (91.6-94.6) | 0.61  (0.29-0.92) |
|  | **-** | 19 | 262 |  |  |  |  |  |  |  |
| **Clinically relevant impulse or conduction abnormality** | | | | | | | | | | |
|  | | **Ref +** | **Ref -** | **Sensitivity** | **Specificity** | **LR+** | **LR-** | **PPV** | **NPV** | **Kappa^1^** |
| **PMcardio** | **+** | 19 | 8 | 70.4%  (49.8-86.3) | 97.0%  (94.1-98.7) | 23.1  (11.2-47.6) | 0.31  (0.17-0.55) | 70.4%  (53.5-83.1) | 97.0%  (94.7-98.3) | 0.98  (0.94-1.00) |
|  | **-** | 8 | 254 |  |  |  |  |  |  |  |

Data are point estimate (95% confidence interval). Reference in each analysis is expert panel consensus on presence of the outcome of interest.

ECG, electrocardiogram; LR+, positive likelihood ratio; LR-, negative likelihood ratio; NPV, negative predictive value; PPV, positive predictive value.

^1^Kappa for interobserver agreement between Android and iPhone.

**Supplementary Table S8.** Validation of the PMcardio app for secondary outcomes in the overall sample (n = 290), assessing only PMcardio assessment with high confidence level as positive for the index test.

| **Significant ST deviation** | | | | | | | | | | |
| --- | --- | --- | --- | --- | --- | --- | --- | --- | --- | --- |
|  | | **Ref +** | **Ref -** | **Sensitivity** | **Specificity** | **LR+** | **LR-** | **PPV** | **NPV** | **Kappa^1^** |
| **PMcardio** | **+** | 2 | 6 | 16.7%  (2.1-48.4) | 97.8%  (95.4-99.2) | 7.7  1.7-34.2) | 0.85  (0.66-1.10) | 25.0%  (7.0-59.7) | 96.4%  (95.5-97.2) | 0.61  (0.29-0.92) |
|  | **-** | 10 | 271 |  |  |  |  |  |  |  |
| **Left ventricular hypertrophy** | | | | | | | | | | |
|  | | **Ref +** | **Ref -** | **Sensitivity** | **Specificity** | **LR+** | **LR-** | **PPV** | **NPV** | **Kappa^1^** |
| **PMcardio** | **+** | 5 | 13 | 45.5%  (16.8-76.6) | 95.3%  (92.1-97.5) | 9.7  (4.2-22.5) | 0.57  (0.33-0.98) | 27.8%  (14.3-47.0) | 97.8%  (96.3-98.7) | 0.88  (0.75-1.00) |
|  | **-** | 6 | 265 |  |  |  |  |  |  |  |
| **Ectopy** | | | | | | | | | | |
|  | | **Ref +** | **Ref -** | **Sensitivity** | **Specificity** | **LR+** | **LR-** | **PPV** | **NPV** | **Kappa^1^** |
| **PMcardio** | **+** | 21 | 5 | 75.0%  (55.1-89.3) | 98.1%  (95.6-99.4) | 39.2  (16.0-95.7) | 0.25  (0.13-0.48) | 80.8%  (63.2-91.1) | 97.3%  (95.1-98.6) | 0.89  (0.79-0.99) |
|  | **-** | 7 | 256 |  |  |  |  |  |  |  |
| **Any BBB** | | | | | | | | | | |
|  | | **Ref +** | **Ref -** | **Sensitivity** | **Specificity** | **LR+** | **LR-** | **PPV** | **NPV** | **Kappa^1^** |
| **PMcardio** | **+** | 20 | 5 | 80.0%  (59.3-93.2) | 98.1%  (95.6-99.4) | 42.2  (17.4-102.9) | 0.20  (0.09-0.45) | 80.0%  (62.2-90.7) | 98.1%  (95.9-99.1) | 0.93  (0.85-1.00) |
|  | **-** | 5 | 259 |  |  |  |  |  |  |  |

Data are point estimate (95% confidence interval). Reference in each analysis is expert panel consensus on presence of the outcome of interest.

BBB, bundle branch block; LR+, positive likelihood ratio; LR-, negative likelihood ratio; NPV, negative predictive value; PPV, positive predictive value.

^1^Kappa for interobserver agreement between Android and iPhone.

**Supplementary Table S9.** Comparison of diagnostic accuracy for the primary and key secondary outcomes of the PMcardio app and the 12-lead ECG device’s automated ECG interpretation algorithm in those with available 12-lead ECG automated interpretation (n = 45).

| **Major ECG abnormalities** | | | | | | | | | | |
| --- | --- | --- | --- | --- | --- | --- | --- | --- | --- | --- |
|  | | **Ref +** | **Ref -** | **Sensitivity** | **Specificity** | **LR+** | **LR-** | **PPV** | **NPV** | **Kappa^1^** |
| **PMcardio** | **+** | 8 | 5 | 72.7%  (39.0-94.0) | 85.3%  (68.9-95.1) | 5.0  (2.0-12.0) | 0.32  (0.12-0.85) | 61.5%  (39.7-79.5) | 90.6%  (78.5-96.2) | 0.31  (0.02-0.59) |
|  | **-** | 3 | 29 |  |  |  |  |  |  |  |
| **12L-ECG AIA** | **+** | 8 | 9 | 72.7%  (39.0-94.0) | 73.5%  (55.6-87.1) | 2.8  (1.4-5.4) | 0.37  (0.14-0.99) | 47.1%  (31.3-63.4) | 89.3%  (75.7-95.7) |  |
|  | **-** | 3 | 25 |  |  |  |  |  |  |  |
| **Atrial fibrillation or flutter** | | | | | | | | | | |
|  | | **Ref +** | **Ref -** | **Sensitivity** | **Specificity** | **LR+^2^** | **LR-^3^** | **PPV^4^** | **NPV^5^** | **Kappa^1^** |
| **PMcardio** | **+** | 7 | 1 | 100.0%  (59.0-100.0) | 97.4%  (86.2-99.9) | 38.0  (5.5-262.9) | - | 87.5%  (50.3-98.0) | 100.0% | 0.73  (0.45-1.00) |
|  | **-** | 0 | 37 |  |  |  |  |  |  |  |
| **12L-ECG AIA** | **+** | 5 | 0 | 71.4%  (29.0-96.3) | 100.0%  (90.8-100.0) | - | 0.29  (0.09-0.92) | 100% | 95.0%  (85.5-98.4) |  |
|  | **-** | 2 | 38 |  |  |  |  |  |  |  |
| **Indication of (past) ischemia** | | | | | | | | | | |
|  | | **Ref +** | **Ref -** | **Sensitivity** | **Specificity** | **LR+** | **LR-** | **PPV** | **NPV** | **Kappa^1^** |
| **PMcardio** | **+** | 2 | 2 | 40.0%  (5.3-85.3) | 95.0%  (83.1-99.4) | 8.0  (1.4-44.9) | 0.63  (0.31-1.30) | 50.0%  (15.1-84.9) | 92.7%  (86.1-96.3) | 0.00  (0.00-0.23) |
|  | **-** | 3 | 38 |  |  |  |  |  |  |  |
| **12L-ECG AIA** | **+** | 1 | 11 | 20.0%  (0.5-71.6) | 72.5%  (56.1-85.4) | 0.7  (0.1-4.5) | 1.10  (0.68-1.78) | 8.3%  (1.5-36.0) | 87.9%  (81.8-92.1) |  |
|  | **-** | 4 | 29 |  |  |  |  |  |  |  |
| **Clinically relevant impulse or conduction abnormality** | | | | | | | | | | |
|  | | **Ref +** | **Ref -** | **Sensitivity** | **Specificity** | **LR+** | **LR-^2^** | **PPV** | **NPV^3^** | **Kappa^1^** |
| **PMcardio** | **+** | 3 | 4 | 100.0%  (29.2-100.0) | 90.5%  (77.4-97.3) | 10.5  (4.1-26.7) | - | 42.9%  (22.8-65.6) | 100.0% | 0.49  (0.11-0.87) |
|  | **-** | 0 | 38 |  |  |  |  |  |  |  |
| **12L-ECG AIA** | **+** | 2 | 2 | 66.7%  (9.4-99.2) | 95.2%  (83.8-99.4) | 14.0  (2.9-67.4) | 0.35  (0.07-1.74) | 50.0%  (17.2-82.8) | 97.6%  (89.0-99.5) |  |
|  | **-** | 1 | 40 |  |  |  |  |  |  |  |

Data are point estimate (95% confidence interval). Reference in each analysis is expert panel consensus on presence of the outcome of interest.

12L-ECG, 12-lead ECG; AIA, automated interpretation algorithm; ECG, electrocardiogram; LR+, positive likelihood ratio; LR-, negative likelihood ratio; NPV, negative predictive value; PPV, positive predictive value.

^1^Kappa for interobserver agreement between PMcardio Android interpretation and the 12-lead ECG automated interpretation algorithm.

^2^Positive likelihood ratio not calculable in absence of true-positives.

^3^Negative likelihood ratio not calculable in absence of false-negatives.

^4^Positive predictive value 95% confidence interval not calculable in absence of true-positives

^5^Negative predictive value 95% confidence interval not calculable in absence of false-negatives
